# Limitations of Plant Stress Tolerance upon Heat and CO_2_ Exposure in Black Poplar: Assessment of Photosynthetic Traits and Stress Volatile Emissions

**DOI:** 10.3390/plants13081165

**Published:** 2024-04-22

**Authors:** Miguel Portillo-Estrada

**Affiliations:** Department of Biology, Research Group PLECO, University of Antwerp, 2610 Antwerp, Belgium; miguel.portilloestrada@uantwerpen.be

**Keywords:** acetaldehyde, acetic acid, heat stress, isoprene, MEP pathway, methanol, MVA pathway, oxidative stress, photosynthesis, stomatal conductance

## Abstract

Volatile organic compounds (VOCs) emitted by plants may help in understanding the status of a plant’s physiology and its coping with mild to severe stress. Future climatic projections reveal that shifts in temperature and CO_2_ availability will occur, and plants may incur the uncoupling of carbon assimilation and synthesis of key molecules. This study explores the patterns of emissions of key VOCs (isoprene, methanol, acetaldehyde, and acetic acid) emitted by poplar leaves (more than 350) under a combined gradient of temperature (12–42 °C) and air CO_2_ concentration (400–1500 ppm), along with measurements of photosynthetic rates and stomatal conductance. Isoprene emission exhibited a rise with temperature and CO_2_ availability, peaking at 39 °C, the temperature at which methanol emission started to peak, illustrating the limit of stress tolerance to severe damage. Isoprene emission was uncoupled from the photosynthesis rate, indicating a shift from the carbon source for isoprene synthesis, while assimilation was decreased. Methanol and acetaldehyde emissions were correlated with stomatal conductance and peaked at 25 °C and 1200 ppm CO_2_. Acetic acid emissions lacked a clear correlation with stomatal conductance and the emission pattern of its precursor acetaldehyde. This study offers crucial insights into the limitations of photosynthetic carbon and stress tolerance.

## 1. Introduction

Volatile organic compounds (VOCs) constitute a diverse spectrum of molecules released into the atmosphere by plants and other organisms. These compounds play pivotal roles in plant’s physiology and its ecological relationships [1,2] They also affect air chemistry and quality by contributing to secondary organic aerosol formation as well as the oxidant power of the atmosphere through the generation of hydroxyl radicals and tropospheric ozone, especially important in urban-like air [3].

Leaf VOC emissions are primarily constitutive, meaning that they are formed either as by-products of metabolic processes associated with plant growth or as a response to tolerable environmental conditions, such as temperature fluctuations, sudden changes in light intensity, transitions from light to darkness [4], oxidative stress-induced accumulation of reactive oxygen species (ROS) [5], and ozone exposure [6]. Given the omnipresence of mild stress, the ability of plants to cope with such conditions is fundamental, highlighting the rarity of an optimal environment for any organism.

On the other hand, non-constitutive emissions are triggered by external agents. For instance, severe abiotic stressors like drought, heatwaves, and high radiation provoke the release of VOCs linked to cell and cell wall damage [7,8], as is the case of methanol emission, generated by pectin demethylation from cell walls upon rupture and exposure to the ambient air. Similarly, VOCs can be released due to the breakage of tissues and vessels resulting from herbivory [9] or the rupture caused by high winds tearing off tissues or breaking branches. Infections by bacteria, fungi, and viruses also induce VOC emissions [10], and recent research has shown connections between emissions and the upregulation of defense genes even in neighboring plants [11,12].

VOC emissions can be categorized either as de novo emissions, where a metabolic process is initiated, leading to the synthesis of new molecules, or as emissions from storage, which include the release of stored compounds, such as the terpenoids stored in resin ducts of coniferous needles or leaf glands [13]. It is noteworthy that de novo emissions are not necessarily constitutive since stressful conditions trigger the emission of, e.g., oxygenated VOCs [5] and hormones [14], and VOC emissions from glands occur constitutively in addition to being stress-induced upon tissue damage. As another example, emissions resulting from programmed leaf senescence are driven by phenology (constitutive), but they exhibit a VOC emission pattern similar to that induced by necrotic tissues during drought (stress-induced) [15,16].

Recent studies emphasize the expanding ecological significance of VOCs, not only within individuals of the same species but also in inter-specific communication and interactions with other organisms [17]. This interconnected network benefits plant fitness and defense, particularly in tri-trophic interactions where a plant attracts the predator of its herbivorous feeder.

VOCs are emitted mainly via stomata [18] and have carbon as fundamental building blocks. Therefore, studying their emission in the context of leaf physiology is especially relevant. For example, elevated levels of atmospheric CO_2_, as well as moderate temperature rise, can increase the availability of carbon in leaves causing greater Rubisco activity and higher rates of photosynthesis [19]. Stomata, crucial for CO_2_ exchange, tend to close under high concentrations of CO_2_ because of an increase in the acidity of guard cells, reducing stomatal conductance (a strategy intended to save water via suppressed transpiration while keeping photosynthetic rates). In this way, under future elevated temperatures caused by elevated CO_2_ levels, plants may incur uncoupling of photosynthetic rates with water transpiration, especially if drought stress is also co-occurring [20]. While moderate warming can stimulate growth, excessive heat can induce stress since Rubisco activity has a range of optimal temperatures after which it decreases, hindering CO_2_ assimilation [21]. Concurrently, rising temperatures influence other metabolic pathways and enzymatic activity, which, coupled with decreased availability of carbon, may alter the rates of biochemical reactions and substrate pools and affect the metabolism [22].

Studying volatile organic compound (VOC) emissions in the context of future air CO_2_ concentrations and air temperatures is of critical importance for several reasons: (1) It helps in the comprehensive assessment of climate change impacts on plant physiology, as alterations in climatic conditions intricately influence plant metabolism shaping the quantity and composition of released VOCs [23]. (2) It allows for assessing the complex dynamics of carbon sequestration and release within ecosystems in response to changing environmental conditions. (3) It allows for assessing potential implications for air quality and human health, particularly concerning VOCs that act as precursors to secondary pollutants [24].

In this article, different temperature levels (ranging from 15 °C to 42 °C) were combined with increasing atmospheric CO_2_ concentrations (ranging from 400 to 1600 ppm) to study the joint responses of plant photosynthesis, stomatal conductance, and the foliar emissions of key VOCs related to primary and secondary production. This allowed for a comprehensive examination of how various potential future scenarios may affect plant metabolic processes.

This article hypothesizes that (1) isoprene, as a VOC that is linked to newly photosynthesized carbon, will exhibit a positive association with photosynthetic activity across all the studied conditions (CO_2_ and temperature combinations). (2) Isoprene emission will increase along with the temperature gradient until a peak emission, where physiological activity will collapse either due to heat stress or by curbed stomatal conductance. (3) The emission of some key oxygenated compounds will exhibit specific patterns, not necessarily similar to that of isoprene, but explained by leaf oxidative stress, occurring, e.g., due to excessive heat.

## 2. Results and Discussion

### 2.1. CO_2_-Mediated Regulation of Isoprene Emissions and Temperature Optima

**Figure 1:**
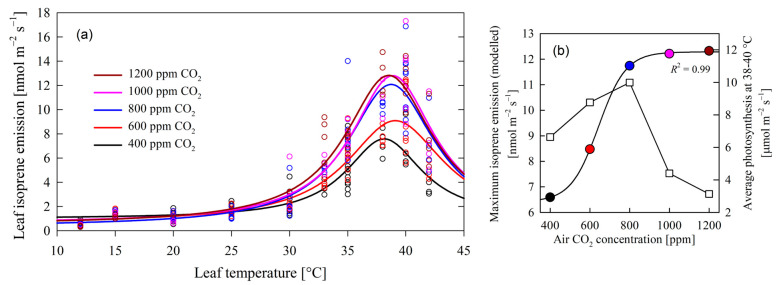
In (**a**), the dependency of leaf isoprene emissions on leaf temperature and increased air CO_2_ concentrations. Maximum isoprene emissions were reached at 38.1–39.1 °C. Emission data series grouped by CO_2_ concentration were fitted to a Lorentzian 4-parameter function, with *R*^2^ values ranging from 0.812 to 0.869. Panel (**b**) depicts the modeled maximum emissions from (**a**) and average photosynthesis at the temperature of peak isoprene emission in relation to air CO_2_ concentration. The data of isoprene emission of (**b**) were fitted to a 4-parameter sigmoid (Equation (1)). Panel (**b**) shows how isoprene emission (circles) increased with CO_2_ concentration, while photosynthesis (squares) dropped at CO_2_ concentrations higher than 800 ppm.

**Figure 2:**
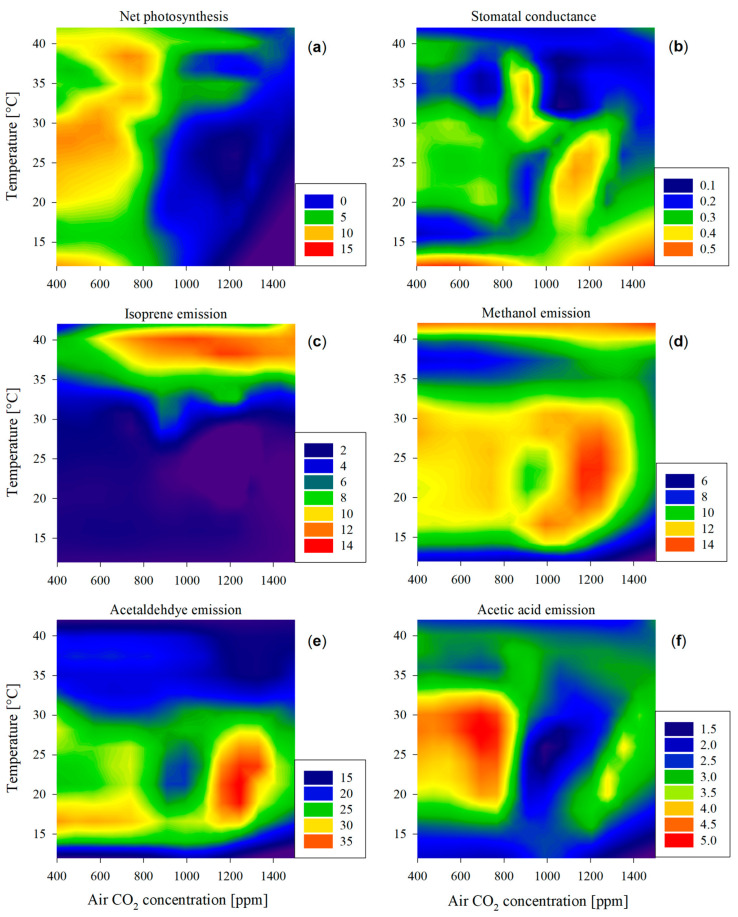
Heatmap illustrating the patterns of (**a**) leaf photosynthesis [μmol CO_2_ m^−2^ s^−1^], (**b**) stomatal conductance [mmol H_2_O m^−2^ s^−1^], and (**c**–**f**) emission patterns of volatile compounds [nmol m^−2^ s^−1^] in response to air temperature (*y*-axis) and CO_2_ concentration (*x*-axis). Higher values for each of the variables are represented in warmer colors (toward red) and lower values with colder colors (toward deep blue), as represented in the legends of each panel.

The analysis of poplar leaf isoprene emissions under varying CO_2_ concentrations and temperature revealed a consistent emission peak at approximately 38.1–39.1 °C (Figure 1), indicative of a temperature optimum for isoprene synthesis after which the emission decreased dramatically. These observations confirmed Hypothesis 2. The decrease could be partially provoked by stomatal closure and low conductivity (Figure 2b) but possibly also by a low capacity to regenerate ribulose-1,5-bisphosphate (RuBP) and inorganic phosphate (Pi) [25,26]. Remarkably, this peak temperature remained stable across the range of CO_2_ levels (400 to 1200 ppm), suggesting that the temperature sensitivity of isoprene peak emissions is independent of CO_2_ concentrations.

The extracted maximum modeled values of isoprene emissions (at 39 °C) showed a notable sigmoidal trend along increasing CO_2_ concentrations (Equation (1), *R*^2^ = 0.99):

(1) $$\Phi_{isoprene}=6.419+\frac{5.866}{1+e^{-\left(\frac{\left[{CO}_{2}\right]-642.9}{69.26}\right)}},$$

where Φ_isoprene_, in nmol m^−2^ s^−1^, is leaf isoprene emission, and [CO_2_], in ppm, is air carbon dioxide concentration (Figure 1b). These findings suggest the following:

(1) As CO_2_ levels increased from Φ_isoprene_ = 6.419 nmol m^−2^ s^−1^, there was a corresponding rise in maximum isoprene emission (Figure 1b), progressing from 6.59 nmol m^−2^ s^−1^ at 400 ppm CO_2_ to 11.74 nmol m^−2^ s^−1^ at 800 ppm CO_2_. Based on this regression, the maximum rise in emissions per unit of [CO_2_] increase was found at 642 ppm CO_2_, with an emission value of Φ_isoprene_ = 9.352 nmol m^−2^ s^−1^ and a maximum rate of emissions of 0.05285 nmol m^−2^ s^−1^ ppm CO_2_^−1^. This positive correlation underlines the influence of CO_2_ concentration on isoprene biosynthesis, aligning with previous studies highlighting the role of carbon availability in secondary metabolite production [27]. Isoprene is produced in the chloroplast primarily from its immediate precursor dimethylallyl diphosphate (DMADP) via isoprene synthase (IspS), which is synthesized via the methylerythritol 4-phosphate (MEP) pathway [28]. Isoprene production is therefore controlled by the supply of DMADP, and by the activity of isoprene synthase [29,30].
(2) Intriguingly, the observed increase in maximum isoprene emission plateaued beyond 800 ppm CO_2_ at about 12.285 nmol m^−2^ s^−1^ (Figure 1b). This suggests a potential saturation point of isoprene synthesis, possibly attributed to limiting enzymatic activity and substrate availability. This stands in contrast to the presumed stimulatory effect of elevated CO_2_ concentrations, which is expected to facilitate de novo carbon assimilation and enhance yields for isoprene synthesis and emissions. Such findings prompt further inquiry into the molecular mechanisms of isoprene biosynthesis and the potential existence of regulatory feedback loops, as found in other poplars subjected to increased levels of [CO_2_] [31].

### 2.2. Controls of CO_2_ Availability and Temperature on Net Photosynthesis

Maximum photosynthetic rates were observed below 800 ppm CO_2_ at temperatures between 25 and 30 °C (Figure 2a) and at higher temperatures at 37–39 °C (Figure 1b). Leaves subjected to CO_2_ levels surpassing 800 ppm experienced a dramatic reduction in net photosynthesis (below 5 µmol m^−2^ s^−1^) at any given temperature (also depicted in Figure 1b at 38 to 40 °C), in line with the results of other studies [32]. This can be attributed to Rubisco downregulation [33] and excessive carbohydrate accumulation in leaves [34].

Stomatal conductance exhibited a notable increase at low temperatures below 15 °C, also observed in poplars previously [35,36] (Figure 2b). Additionally, significant peaks in stomatal conductance were observed under the conditions of elevated CO_2_ concentrations ranging from 800 to 1200 ppm and a temperature range of 20 to 35 °C. Photosynthetic activity and stomatal conductance did not exhibit a parallel trend throughout the observed conditions. In particular, when high stomatal conductance values were associated with elevated CO_2_ levels, carbon assimilation remained low. This discrepancy likely indicates an inefficient trade-off between the loss of leaf water and CO_2_ intake for photosynthesis. These findings emphasize the need for further investigation of water use efficiency since it has implications for plant water status and carbon assimilation during transient elevated CO_2_ conditions or under a climatic change situation where such levels could be met.

The present investigation was conducted under controlled conditions, ensuring uniformity in light exposure, temperature, and ambient CO_2_ concentration for the studied plants. Further experiments with entire trees grown under distinct combinations of temperature and ambient CO_2_ concentration should be conducted to more accurately replicate the responses of poplars to future climate scenarios.

### 2.3. Impairment of Carbon Assimilation and Isoprene Emission

Isoprene emission remained almost constantly low at temperatures below 30 °C across all CO_2_ concentrations (Figure 1a and Figure 2c). It only peaked at temperatures from 35 to 40 °C at all air CO_2_ concentrations (Figure 1a), with the exception of 400 ppm CO_2_, where isoprene emission was significantly smaller (Figure 1b).

Crossing these observations with the net photosynthesis pattern (Figure 1b and Figure 2a), there was an impairment between maximum isoprene emissions and net photosynthesis: Under the same light availability conditions, photosynthesis exhibited high rates across the range of temperatures studied and below 800 ppm CO_2_, indicating a relatively robust trend (Figure 2a); in contrast, isoprene emissions demonstrated a distinct pattern, exclusively peaking at elevated temperatures exceeding 35 °C, increasing in the range of 400–800 ppm, and then stabilizing at higher CO_2_ levels (Figure 1). In short, at high temperatures, elevated carbon assimilation co-occurred with high isoprene emission only under CO_2_ levels not higher than 800 ppm (Figure 1b).

Firstly, this suggests that, at higher CO_2_ levels where photosynthesis is inactive, isoprene synthesis derives its carbon from a source other than newly photosynthesized carbon [37]. Under these conditions, isoprene emission could potentially be sustained as a result of the synthesis via the mevalonic acid (MVA) pathway, which is an alternative pathway to the previously mentioned MEP pathway for isoprene biosynthesis. It uses carbon from acetyl-CoA, typically via glycolysis, fatty acid degradation, and amino acid catabolism [38].

Secondly, the impeccable sigmoidal trend found in Figure 1b indicates that isoprene emission is sustained (at 38–39 °C) as carbon assimilation starts declining. More research is needed to investigate whether the activation of the MVA pathway occurs upon the shutdown of the MEP pathway or whether both co-exist and there is a smooth transition in their activity level toward the MVA pathway to sustain isoprene synthesis. This potential transition could reveal the mechanism through which plant species manage to tolerate high temperatures, and it could explain the lack of carbon assimilation under increased CO_2_.

Thirdly, further research could enable the prediction of what would be the emissions of isoprene under possible future CO_2_ concentrations. Isoprene synthesis has been shown to help scavenge reactive oxygenated species (ROS) generated at high temperatures and stabilize the thylakoid membrane in order to avoid cell lysis and reduce the formation of reactive oxygen species [39]. Despite certain tolerance to the stress described, isoprene synthesis had a limitation that could lead to metabolic dysfunction in the leaf. The use of inhibitors to favor one or another synthetic pathway, as well as the information on maximum emissions and the slope of Equation 1 found in this article, can elucidate the actual speed of the MEP and MVA pathways along the gradient of CO_2_ concentrations and their precise activation/deactivation points.

An attempt to model the isoprene emissions at temperatures between 20 and 38 °C (the last measurement before the drop after the observed peak emission) was made using CO_2_ concentration, temperature (*T*_a_), net photosynthesis, stomatal conductance, and chlorophyll content. The use of the first two variables provided a multiple linear model with satisfactory results proving Hypothesis 1, *R*^2^ = 0.83, *p* < 0.001 (Equation (2)):

(2) $$\Phi_{isoprene}=e^{-2.854+0.000354\times \left[{CO}_{2}\right]+0.125\times T_{a}}.$$

It is important to note that isoprene can be also considered a secondary metabolite (terpenoid). In that case, the enhanced emission can occur after a prolonged period of growth inhibition, and its carbon is sourced from reduced carbon that was previously assimilated and stored [40]. Therefore, an immediate response of isoprene emission with a change in the photosynthetic rate cannot be always expected.

In the current study, the exposure to environmental conditions indicated that different carbon sources for isoprene synthesis could sustain the elevated emissions upon photosynthetic shutdown. Therefore, a further setup consisting of gradual changes—lasting over hours, such as diurnal trends—in both temperature and CO_2_ concentration and the monitoring of VOC emissions could unveil valuable insights into the regulation of isoprene synthesis.

### 2.4. Emission of Oxygenated Compounds under Different Temperatures and CO_2_ Levels

A prominent peak in the emission levels of oxygenated compounds was found for both methanol and acetaldehyde at around 25 °C and 1200 ppm CO_2_ (Figure 2d,e) and their general emission patterns across temperatures and CO_2_ levels were different from the pattern of photosynthetic activity, confirming Hypothesis 3. The high emissions under the mentioned conditions could possibly be related to a higher stomatal conductance (Figure 2b) since water-soluble compounds—typical components of many oxygenated VOCs—can be particularly strongly controlled by stomatal conductance due to high temporal storage in the leaf liquid phase [41,42]. Both compounds were emitted at mid-range levels, while leaves were subjected to mid-range temperature and 400–800 ppm CO_2_. In fact, a linear correlation between the emission of methanol and acetaldehyde was found (Equation (3); *r*^2^ = 0.712, *p* < 0.001) in temperatures between 15 and 30 °C:

(3) $$\Phi_{acetaldehyde}=4.041\times \Phi_{methanol}+18.66.$$

The correlation included measurements at optimal conditions (400 ppm and 25 °C) where acetaldehyde emission was low in comparison to methanol. While a moderate level of methanol emission at optimal growing conditions is related to the demethylation of pectins in cell walls during leaf tissue growth [43], acetaldehyde was not expected to be emitted in such amounts. This is because high acetaldehyde emission is more likely related to stress-related conditions, such as the decarboxylation from pyruvate (pyruvate over-flow mechanism) [44] and the oxidation of xylem-transported ethanol [45], typically occurring during anaerobic conditions, e.g., root flooding. These two situations were unlikely in the current study. In conclusion, the large emissions of acetaldehyde at around 25 °C and 1200 ppm cannot be explained either by increased stomatal conductance (Figure 2b,e) refuting the part of the third hypothesis. Moreover, no damage to the leaves was observed in the study, suggesting that the peak emissions of acetaldehyde were not related to leaf wounding [46].

Methanol emission was also elevated at high temperatures, regardless of CO_2_ emissions (Figure 2d), overlapping with the dramatic drop in isoprene emission after its peak at about 38.1–39.1 °C (Figure 2c). In this phenomenon, we could observe the limit of stress tolerance since a higher temperature than the isoprene synthesis peak affected the leaf physiology dramatically in several steps: Stomatal conductance was minimal; the leaf could not maintain photosynthesis; isoprene could not help stabilize cell membranes; and cell wall starting breaking, producing methanol as a result of pectin demethylation [47]. In this case, methanol emission was not related to leaf transpiration, and given the low stomatal conductance at temperatures above 40 °C, it could be that the intracellular concentration of methanol increased to a much higher degree than what can be extrapolated from its emission levels. All these phenomena and possible explanations prove Hypothesis 3.

Acetic acid emissions were high in the range from 25 to 30 °C and from 400 to 800 ppm. As explained previously, acetic acid is derived from the oxidation of ethanol and acetaldehyde within leaves [15]. However, despite this tight relationship and its water solubility that should lead to a correlation with stomatal conductance, there was no robust relationship between these two variables, similar to what was found in a year-long study at a poplar plantation [48]. More investigation is needed on the controls of the emission of acetic acid, especially because it did not exhibit a maximum at mid-range temperatures and higher CO_2_ availability, as would be expected. Further investigation will clarify whether the emission patterns are, as hypothesized, more related to the stressful conditions of oxidative stress.

## 3. Materials and Methods

### 3.1. Plant Material

*Populus nigra* L. (Brandaris genotype) clone cuttings, originated from the Lochristi research site (51°06′39″ N 3°50′57″ E), were planted in 20 L pots, in a soil composed of 10% peat and 90% sand in volume. The trees were grown from these cuttings at a field site within the University of Antwerp Campus Drie Eiken in Belgium (51°09′42″ N, 4°24′31″ E) for two years, always cutting eventual root suckers and leaving one main stem. Annually, the trees received an optimal amount of fertilizer, with a total of 70 g NPK (De Ceuster Meststoffen DCM Ecor NPK 8-5-6) and 3.6 g of micronutrients (DCM micro-mix) per pot. At the beginning of the growing season of the third year, the twelve best-fitted trees were transferred to a growing chamber indoors for subsequent measurements. The shoots were cut back to allow new shoots to grow from the tree stump in the new growing conditions. The climate chamber was operated at 16h photoperiod under growing lamps (LED grow light bar Mezzo 85W, TotalGrow, Alvin, TX, USA), temperature 22 °C/16 °C cycle for day and night, 400 ppm CO_2_, and relative humidity ca. 40–60%. The pots were regularly irrigated to keep the soil moist while avoiding building a water table. At the time of the experiment, the trees were perfectly acclimated, and all the leaves used for the measurements were grown to maturity under the described environmental conditions.

We used fully expanded leaves alternating the trees and the shoots to avoid stressing a single individual at a time and allowing for randomness in the measurements.

### 3.2. Analytical Setup

Leaf photosynthetic activity, stomatal conductance, and volatile organic emissions were measured online using a LI-COR 6400XT (Li-Cor, Lincoln, NE, USA) combined with a proton-transfer-reaction time-of-flight mass spectrometer (PTR-TOF-MS) model 8000 (Ionicon Analytic GmbH, Innsbruck, Austria). The clip-on-type leaf cuvette covered 6 cm^2^ of the leaf surface, and the enclosed leaf area was illuminated with an LED array/PAM-fluorometer 3055-FL. The optimal amount of photosynthetically active radiation was determined by performing regular light/photosynthesis curves on the leaves used, ranging from 1200 to 1600 μmol photon m^−2^ s^−1^. The chamber was operated at a flow rate of 500 μmol s^−1^ and a constant air humidity of ca. 60%. The chamber block temperature and air CO_2_ concentration were controlled via the Li-Cor instrument.

The conditions used during the experiment were 12, 15, 20, 25, 30, 35, 40, 42 °C, and 400, 600, 800, 1000, 1200, 1400, and 1500 ppm CO_2_, amounting to 56 possible combinations. More than 350 leaves were used in the experiment, each assigned to a single combination of temperature and CO_2_ concentration, yielding 5 to 8 replicate leaves per combination of CO_2_ and temperature.

The emission of several VOCs was monitored due to their proven implication in diverse physiological processes of interest, i.e., isoprene (C_5_H_8_H^+^, *m*^+^/*z* = 69.070) for photosynthetic activity [49], methanol (CH_4_OH^+^, *m*^+^/*z* = 33.034) for cell wall growth [50] and cell wall degradation [9], and acetaldehyde (C_2_H_4_OH^+^, *m*^+^/*z* = 45.033) and acetic acid (C_2_H_4_O_2_H^+^, *m*^+^/*z* = 61.028) for their relation to oxidative stress [5,8].

A constant flow of 74 μmol s^−1^ exiting the leaf cuvette outlet was diverted to a PTR-TOF-MS instrument. The drift tube was operated at 600 V, 2.5 mbar pressure, and 60 °C temperature, resulting in a field density ratio (E/N) of ≈120 Td. PTR-ToF-MS raw data were recorded by the TofDaq data acquisition software (Tofwerk AG, Thun, Switzerland). The protonated ions were extracted at a 32 µs rate into the time-of-flight region. The data were recorded at 1 s time resolution, resulting from the average of 31,250 spectra (*m*/*z* 1–316). More details about the instrument operation, calibration, and raw data processing to calculate VOC concentrations are found in a previous study that applied the same PTR-TOF-MS measurement setup [46], as well as the peak-fitting details for the ions and the discrimination of adjacent peaks in the spectrum [48]. TofViewer software (version 3.4.4, Ionicon Analytik, Innsbruck, Austria) was used for data postprocessing. The peaks in the spectrum were fitted to a Gaussian function on its left side and to a Gumbel function on its right side. The coefficients of reaction between each VOC and H_3_O^+^ were retrieved from the study by Cappellin et al. [51]. The natural abundance ratios of the parent ions containing ^1^H, ^12^C, and ^16^O were 98.6414% (methanol), 97.5938% (acetaldehyde), 97.3568% (acetic acid), and 94.7041% (isoprene).

All equation fittings were performed in Sigmaplot v14.5 for Windows (SPSS Inc., Chicago, IL, USA). The data in Figure 2 are presented as a heatmap using the filled contour graph tool from the software.

## 4. Conclusions

The findings of this study emphasize a robust association between air temperature, CO_2_ concentration, and VOC emissions, particularly isoprene, methanol, acetaldehyde, and acetic acid. Notably, a consistent peak in isoprene emission occurred at 38–39 °C, further exacerbated by elevated CO_2_ levels, with emissions reaching a plateau beyond 800 ppm CO_2_. The observed sigmoidal relationship between maximum isoprene emission and CO_2_ concentration suggested a distinct carbon source beyond the direct photosynthetic pathway at higher CO_2_ levels, prompting the need for further exploration of underlying mechanisms. Methanol and acetaldehyde emissions peaked at 25 °C and 1200 ppm CO_2_, exhibiting a positive correlation with stomatal conductance. In contrast, acetic acid emissions, tied to acetaldehyde oxidation in the existing literature, displayed a less defined correlation with stomatal conductance and the emission pattern of its precursor. These findings contribute to a deeper understanding of the complex interplay between environmental variables and volatile organic compound emissions, offering insights into the constraints on plant stress tolerance under heat and CO_2_ exposure.

## Data Availability

The original data presented in the study are openly available at https://zenodo.org/doi/10.5281/zenodo.11029439 (accessed on 19 April 2024).
